# Comparing college students’ mood states among immersive virtual reality, non-immersive virtual reality, and traditional biking exercise

**DOI:** 10.1371/journal.pone.0311113

**Published:** 2024-11-21

**Authors:** Wenxi Liu, Daniel J. McDonough, Zan Gao

**Affiliations:** 1 Department of Physical Education, Shanghai Jiao Tong University, Shanghai, China; 2 School of Public Health, University of Minnesota, Minneapolis, Minnesota, United States of America; 3 Department of Kinesiology, Recreation, and Sport Studies, The University of Tennessee, Knoxville, Tennessee, United States of America; Qatar University College of Education, QATAR

## Abstract

**Objectives:**

This study examined differences in young adults’ mood states during immersive virtual reality (VR), non-immersive VR, and traditional exercise biking sessions.

**Design:**

Repeated-measure study design.

**Methods:**

Forty-nine college students (34 females; M_age_ = 23.6 years) completed three separate 20-minute exercise biking sessions: (1) immersive VR biking using the PlayStation 4 + VirZoom VR bike; (2) non-immersive VR biking using the Gamercize bike + Xbox 360; and (3) traditional stationary biking using the Spirit Fitness XBU55. Participants’ mood states (anger, confusion, depression, fatigue, tension, and vigor) were assessed by using the Brunel Mood Scale after each session.

**Results:**

Statistically significant differences were observed between biking sessions for all components of mood [F (2, 96) = 3.84–278.56, *p* < 0.05, η^2^ = 0.07–0.85], except for tension (*p* > 0.05). Results indicated non-immersive VR biking yielded significantly higher anger compared to immersive VR biking; non-immersive VR biking yielded significantly higher confusion compared to immersive VR biking and traditional biking, respectively; immersive VR biking yielded significantly lower depression compared to traditional biking; both immersive VR biking and non-immersive VR biking yielded significantly lower fatigue compared to traditional biking; and immersive VR biking yielded significantly higher vigor compared to non-immersive VR biking) and traditional biking, respectively.

**Conclusion:**

Findings suggested the immersive VR-based biking exercise may facilitate in reducing the negative feelings, such as anger, fatigue, depression, and improving positive feeling, such as vigor, among college students. The immersive VR-based exercise appeared to be a feasible approach for motivating college students participating in physical activity and improving overall mood states.

## 1. Introduction

Considerable research evidence has indicated that regular participation in physical activity (PA) leads to improvement in both physical and mental well-being [1]. Physical benefits include reducing the risk of overweight and obesity, improving cardiovascular fitness and cholesterol profiles, and decreasing the risk of developing type 2 diabetes [2]. Mental health benefits include reducing symptoms of anxiety and depression and improving affective states (e.g., mood states) [3, 4]. Recently, there has been a growing public health concern regarding the declined PA and increased mental health issues in the U.S. college student population due to their newfound responsibilities of balancing school, work, and personal responsibilities. According to the National Alliance of Mental Illness, nearly 80% of college students feel overwhelmed by their academic responsibilities and about 30% reported having problems with college work due to mental health issues [5]. Moreover, 44% of U.S. college students reported having symptoms of depression. In addition, the National College Health Assessment reported only about 20% of college students met the recommended PA guidelines of at least 150 minutes of moderate-to-vigorous intensity PA (MVPA) per week [6]. Previous research has indicated that the positive changes in mood state could take place in as little as 10 minutes of moderate-intensity exercise [7]. Despite the known benefits, college students often fail to sustain sufficient PA due to a lack of interest or motivation to do so [8]. Compared to monotonous and repetitive PA programs, college students may prefer leisure activities after a long day of work or school, such as playing video games or watching TV, where entertainment can be obtained while relaxing [9]. However, most leisure time activities (e.g., video game playing, screen viewing) have been contributed to the high prevalence of sedentary behavior among this population. Fortunately, as the technology advances, virtual reality (VR)-based active video games allow users to play games while exercising. With greater interaction, immersion, and fun features, VR-based exercise may trigger college students’ interest and motivation toward PA and improve their physical and mental well-being [9–13].

VR is an interactive, computer-generated experience taking place within a simulated environment, with this technology primarily incorporating auditory and visual feedback [14]. VR-based exercise establishes the connection between a virtual environment and actual movement. Individuals who exercise on a VR-based exercise apparatus are able to interact with the virtual gaming environment by exercising in the real world. Generally, there are two types of VR: immersive VR and non-immersive VR. In detail, immersive VR utilizes a head-mounted apparatus, body motion sensor, real-time graphics, and an advanced interface device to simulate the complete virtual environment which envelopes players in a virtual world. Non-immersive VR uses a flat computer/television screen linked to a keyboard, gamepad, and joystick to interact with the gaming system [15]. As compared to non-immersive VR, immersive VR allows individuals to sense a greater level of “presence” – the subjective feelings of being in the virtual environment [16]. As demonstrated by the previous research, individuals may experience more tensive and realistic emotional reactions, such as fear or joy, as the perception of presence increased [17]. Therefore, many clinical studies have adopted such technology as an approach for psychotherapy [18]. For instance, phobia patients can be safely exposed to their fears in the virtual environment and still experience similar emotional reactions to those in reality. Similarly, if individuals exercise in a virtual environment and sense a greater level of presence, their mood states can also be influenced by the experiences that they encountered within it [19]. However, the effects of immersive VR-based exercise on college student’s mood states have been rarely studied.

In the past decade, VR technology has been primarily adopted in rehabilitation and clinical fields, such as facilitating post-stroke patients’ balance and mobility training and alleviating phobia patients’ symptoms by training in the virtual environments [20]. Recently, VR has become common to general population as more commercial VR apparatuses are available, such as Meta Quest, PlayStation VR, PICO VR, HTC Vive, etc. Given the popularity of VR, researchers and health professionals have begun to explore its potential in promoting physiological and psychological health among healthy populations [21, 22]. Previous studies indicated that exercise in a visually stimulating environment may lead to greater improvements in self-efficacy and mood compared to less stimulating environments [23, 24]. In addition, Plante and colleagues conducted an experiment to examine whether the combination of VR and exercise enhance individuals’ psychological health (i.e., mood states) [25]. Participants were randomly assigned to one of three 30-minute conditions: (1) riding a stationary bike at moderate intensity; (2) playing VR bike game without exercising; and (3) playing a VR bike game while riding on a stationary bike. The results suggested the combination of VR and exercise may improve mood states, such as enjoyment, energy, and reduced perception of tiredness. Another study which combined VR and stationary cycling observed participants exercising with VR feedback reported lower perceived exertion and higher enjoyment compared to exercise alone [21]. The findings indicated that exercising in VR environments may attract individual’s attention from body movement to virtual environment interactions which may potentially enhance the duration of a given PA session. Given the fun and unique nature of VR-based exercise and the demonstrated positive effects psychological well-being, VR-based exercise may be considered as a potentially effective approach to attract young adults to be physically active while improving their overall psychological well-being [26, 27].

Based on available studies, the immersive VR-based exercise may serve as an effective alternative for improving overall psychological states [9, 11, 14, 20, 25, 28]. As an innovative technology, the use of immersive VR-based exercise as an approach to improve college students’ mood states and psychological well-being has been rarely studied. Therefore, the purpose of this study was to examine the effects of immersive VR-based biking exercise on college students’ mood states in comparison with non-immersive VR and traditional biking exercises. The findings of this study would provide insights and practical implication for using immersive VR-based exercise as a means promoting PA and mental health among young adults.

## 2. Materials and methods

### 2.1 Participants

A total of 49 college students were recruited from an urban public university in the Midwest region of the U.S. Participants were recruited via flyers posted and word of mouth on campus from 03/09/2019 to 10/5/2020. The study inclusion criteria included: (1) 18–35 years old currently enrolled college students; (2) no self-reported diagnosed physical or mental disability; (3) successful completion of the Physical Activity Readiness Questionnaire [29]; (4) no self-reported motion sickness to VR-based exercise; and (5) provision of informed consent for participation. Prior to any data collection, this study was approved by the University’s Institutional Review Board (STUDY00006005) and all procedures were followed in accordance with the ethical standards of the Institution. All participants have provided signed consent to participate in this study. Notably, participants’ previous VR experiences were not assessed in this study.

### 2.2 Study settings

We employed a repeated-measure design, and the study was conducted in a well-established and highly controlled laboratory. Participants were asked to exercise for three separate 20-minute sessions in a random and counterbalanced order: (1) immersive VR exercise bike (VirZoom, Cambridge, MA, U.S.); (2) non-immersive VR exercise bike (Gamercize Bike, Southampton, U.K.); and (3) traditional exercise bike (Spirit Fitness 156 XBU55 Upright Bike, Spirit fitness, Jonesboro, AR, U.S.).

The immersive VRbiking provides a fully immersive virtual gaming environment by connecting the exercise bike and PlayStation VR system. Two VR exergames were employed during the 20 minutes VR biking session. The first exergame, "Le Tour," had players race against virtual cyclists, navigating through a series of timing gates on a picturesque mountain road. In the second exergame, "Race Car," participants raced virtual cars around a track at high speeds. For both games, players needed to pedal faster or slower to control their speed, and lean their torsos side-to-side to turn left or right during gameplay. In this session, participants were exercising under fully immersive virtual environment by wearing a head-mounted display (HMD). The immersive VR HMD is capable of providing users with a wider field-of-view than flat screen which may elicit higher level of immersion [30].

The Gamercize exercise bike was utilized for the non-immersive VR condition and connected to an Xbox 360. Participants engaged in the game "Motocross," where they navigated a racecourse against virtual competitors. Using a standard Xbox 360 controller, they steered the motocross bike. Notably, participants needed to maintain a pedaling cadence above 60 rotations per minute; otherwise, the controller would shut off, stopping the gameplay. In this session, participants were interacting with virtual environment via a flat screen. When comparing with immersive VR, non-immersive VR offers relatively lower level of immersion in the virtual environment.

The Spirit Fitness XBU55 upright stationary bike was employed for the traditional cycling session. Both the immersive and non-immersive VR cycling sessions maintained an exercise intensity equivalent to 65% to 85% of the age-predicted maximum heart rate. To ensure consistency across all three cycling sessions, participants were required to keep their heart rate within this range, monitored by the bike’s built-in heart rate sensor during the traditional cycling session.

### 2.3 Measurements

Participants’ age, sex, and race/ethnicity were obtained from a self-reported demographic questionnaire. Height was measured to the nearest 0.5 cm using a Seca stadiometer (Hamburg, Germany). Weight and body fat percentage were assessed via the Tanita BC-558 IRONMAN® Segmental Body Composition 164 Monitor (Tokyo, Japan). The body mass index (BMI) was calculated by the weight divided by the square of height (kg/m^2^).

Participants’ mood states were assessed using the Brunel Mood Scale (BRUMS) [31]. In detail, the BRUMS includes six subscales that measure different mood states—tension, depression, anger, vigor, fatigue, and confusion—each with four items. Participants selected each item from a Likert-type scale of zero to four (0 = *not at all*, 1 = *a bit*, 2 = moderate, 3 = *enoug*h, 4 = *extremely*), which they had been feeling in the moment of assessment. Sample items of tension included “annoyed, bitter, angry, bad-tempered”, sample items of confusion included “confused, muddle, mixed-up, uncertain”, sample items of depression included “depressed, downhearted, unhappy, miserable”, sample items of fatigue included “worn out, exhausted, sleepy, tired’, sample items of tension included “panicky, anxious, worried, nervous”, and sample items of vigor included “lively, energetic, active, alert”. Participants were asked to complete this survey immediately following each biking exercise session. The subscales’ mean scores were calculated and used as the primary outcomes.

### 2.4 Procedure

Participants were asked to exercise on three 20-minute biking session in a random and counterbalanced order. Breaks between each biking session were 10 minutes in duration and allowed for participants’ blood pressure and heart rate to return to baseline levels. During each break, participants were asked to complete survey assessing current mood states.

### 2.5 Statistical analyses

First, data were screened for outliers using a boxplot and normality of distributions using the Shapiro–Wilks tests of normality. Second, descriptive statistics were used to describe participants’ characteristics and study outcomes. Third, repeated measures ANOVA was used to examine the differences in mood states (i.e., tension, depression, anger, vigor, fatigue, and confusion) between three biking exercise sessions. All analyses were performed using IBM SPSS 25.0 (Armonk, NY, USA). The significant level was set to p < 0.05. The effect size was calculated as partial eta squared (η^2^).

## 3. Results

A total of 49 college students (see Table 1) completed three biking exercise sessions. Overall, significant differences (see Table 2) were observed between three biking sessions for participants’ mood states [F (2, 96) = 3.84–278.56, *p* < 0.05, η^2^ = 0.07–0.85], except for tension (*p* > 0.05). Post hoc Bonferroni comparisons further revealed that immersive VR had significantly lower anger compared to non-immersive VR (1.5 ± 0.66 vs. 1.09 ± 0.21); non-immersive VR had significantly higher confusion compared to immersive VR (1.51 ± 0.69 vs 1.26 ± 0.53) and traditional biking (1.51 ± 0.69 vs. 1.20 ± 0.4), respectively; immersive VR had significantly lower depression compared to traditional biking (1.07 ± 0.18 vs 1.34 ± 0.68); both immersive VR (1.86 ± 0.72 vs. 2.47 ± 0.87) and non-immersive VR (1.81 ± 0.74 vs 2.47 ± 0.87) had significantly lower fatigue compared to traditional biking; immersive VR had significantly higher vigor compared to non-immersive VR (3.70 ± 0.93 vs. 1.30 ± 0.47) and traditional biking (3.70 ± 0.93 vs. 1.15 ± 0.38), respectively.

**Table 1:** Demographic characteristics of participants.

	*M*	*SD*
Age (yr)	23.61	3.39
Height (m)	1.69	0.80
Weight (kg)	68.25	13.90
BMI	23.81	3.56
%BF (%)	23.97	7.46

Note: M, mean; SD, standard deviation; BMI, body mass index; %BF, percentage of body fatness.

**Table 2:** Average score of mood states outcomes within each exercise session.

	Anger		Confusion		Depression		Fatigue		Tension		Vigor	
	*M*	*SD*	*M*	*SD*	*M*	*SD*	*M*	*SD*	*M*	*SD*	*M*	*SD*
Immersive VR Bike	1.09	0.21	1.26	0.53	1.07	0.18	1.86	0.72	1.24	0.39	3.7	0.93
Non-Immersive VR Bike	1.46	0.66	1.51	0.69	1.28	0.64	1.81	0.74	1.29	0.47	1.29	0.47
Traditional Bike	1.31	0.67	1.2	0.41	1.35	0.68	2.47	0.87	1.15	0.38	1.15	0.38

Note: M, mean; SD, standard deviation.

## 4. Discussion

The purpose of this study was to examine the differences regarding young adults’ mood states among immersive VR, non-immersive VR and traditional biking exercises. Overall, the findings of this study support the notion that immersive VR-based exercise may lead to greater improvement in colleges students’ mood states as compared to non-immersive VR and traditional biking exercises [27]. Specifically, participants in the immersive VR exercise condition reported greater vigor and lower anger, confusion, and fatigue compared with non-immersive VR and traditional exercise. Further, immersive VR exercise provided participants with greater immersion and a fun and unique exercising experience which may attract more college students to spend more time being physically active and reduce time spent in sedentary behaviors and thus, obtaining psychological benefits to attenuate the stress from balancing school, work, and personal responsibilities.

The results of this study indicated participants exercising in the fully immersive VR biking condition reported the highest vigor as compared to the other two conditions. Vigor refers to the sense of being lively, energetic, active, and alert [32]. This finding was in line with previous studies which indicated the combination of VR and exercise lead to improvements in favorable psychological outcomes [9, 10, 25, 28]. Plante and colleagues conducted a cross-sectional study which examined the psychological benefits of VR-based exercise among 154 college students [28]. Participants were randomly assigned to one of four conditions for 20 minutes: (1) walking around campus; (2) walking on a treadmill and wearing a VR head-mounted display; (3) walking on a treadmill without VR; or (4) experiencing a virtual walk by VR without actual walking. The investigators found increased perception of vigor and mood benefits immediately after exercising with and without VR conditions. This supports the notion that psychological well-being benefits could be observed immediately after exercise [7, 33]. It appears that the immersive VR exercise is capable of inducing improved energy levels with a more enjoyable exercising experience. Offering high level of immersion is one noticeable feature of immersive VR. Immersion reflects individual’s psychological feeling of being in the virtual environment [34]. The VR HMD envelops users into a replicated real word and reflects physical movements in the virtual environment, leading to a sense of “presence”. Studies have shown that higher level of immersion was associated with increased arousal under VR condition [35]. Corresponding to our study, the higher level of immersion and gaming interactions in the immersive VR biking may draw participants’ attention then increase their feeling of vigor. However, our study did not use scale to measure the level of immersion, future studies are recommended to adopt scale assess the level of immersion and further investigating the underlying mechanism regarding immersive VR exercise promoting positive psychological well-being.

In addition, the findings indicated that participants in the immersive VR exercise condition reported lower perception of fatigue as compared to the other conditions. Fatigue refers to the perception of being worn-out, exhausted, sleepy, and tired [36]. As college students experienced less fatigue when exercising on the immersive VR bike, it is possible that they may want to exercise for a longer duration. Zeng et al in a pilot study, examined the effects of immersive VR exercise biking on college students’ physiological and psychological outcomes [14]. The study found participants on the immersive VR bike had higher levels of enjoyment, self-efficacy, and lower levels of perceived exertion. Moreover, McDonough and colleagues also observed lower perceived exertion when exercising in a VR-based condition as compared to traditional exercise [11]. It appears that the findings of less perception of fatigue or exertion on VR-based exercise is consistent across studies. The potential explanation for this finding may attribute to the higher level of immersion and VR exergaming interactions during immersive VR biking, which lead to more focused on the VR biking session and distract their attention from exercise-induced fatigue. The fun nature of VR exergames and gaming interactions attract players’ attention and motivate them playing while exercising. In addition, the gaming flow and the progressive challenges in Immersive VR exercise may motivate participants to engage in more PA during the session. Overall, immersive VR exercise appears to have the potential to serve as an alternative exercise +-means motivating young adults participating in PA. However, all the preceding studies were cross-sectional design, the long-term of impact of immersive VR exercise with longitudinal designs are needed.

Concerning the mood states between immersive VR and non-immersive VR biking sessions, participants reported relatively lower confusion and anger in immersive VR session as compared to the non-immersive VR session. A potential explanation of this may explain by the different gaming experiences. The non-immersive VR bike required participants to keep pedaling at certain pace while using a wired controller to play the game. If participants’ pedaling cadence fell under 60 rotations per minute, the game would shut down. This might be a challenge for some participants racing with avatars in the game while maintaining the pedaling cadence. This may have resulted in more confusion. Contrarily, the gaming experience during the immersive VR bike seemed much easier in that the participants were able to control the game using their body movements by leaning side-to-side. As immersive VR technology becomes more and more popular, future studies may consider adopting such types of VR as the exercise modality.

Taken together, the findings of this study provided preliminary empirical evidence that the use of immersive VR-based biking exercise may be a feasible approach to promote college students’ PA [37] while improving their overall mood states. To the best of our knowledge, this was the first study to compare the acute effects of VR biking exercise on heathy college students’ mood states as compared to non-immersive VR and traditional biking exercise. Moreover, while most of the immersive VR-based studies have been based in clinical settings, the present study provided insights of its utility for improving mood states among general population. Nevertheless, limitations of this study warrant future researchers’ consideration. First, the mostly female and non-Hispanic White sample may hinder the ability to identify sex and/or racial differences regarding study outcomes. Indeed, Plante et al., in their VR-based treadmill walking study, observed female participants to have greater emotional reactions than males when exercising under in virtual environments [4, 28]. Future studies may also consider the potential gender differences regarding the assessed psychological outcomes. Second, the cross-sectional study design does not allow us to draw conclusions of causal evidence. In light of these findings, future randomized control trials are warranted. Third, although we adopted random and counterbalanced order and 10-minute between-session breaks, the carry-over effect may potentially affect the study outcomes, such as mental fatigue. Future studies may adopt independent groups to examine the outcome differences.

## 5. Conclusions

The findings of this study suggested immersive VR-based biking exercise may facilitate in reducing the negative feelings, such as anger, fatigue, depression, and improving positive feeling, such as vigor, among college students. The immersive VR-based exercise appeared to be a feasible approach for motivating college students participating in physical activity [38] and improving overall mood states.
